# Supplementary material for: Association between dietary calcium, potassium, and magnesium consumption and glaucoma
Source: PLoS One. 2023 Oct 18;18(10):e0292883. doi: 10.1371/journal.pone.0292883 (PMC10584168; doi:10.1371/journal.pone.0292883)
Supplement: S1 Table — (DOCX) [file pone.0292883.s002.docx]

**Table S1. Description of missing variables**

| Variables | N (%) |
| --- | --- |
| Education level | 4 (0.06) |
| Marital status | 3 (0.05) |
| PIR | 455 (7.35) |
| Eye surgery for nearsightedness | 255 (4.12) |
| Eye surgery for cataracts | 247 (3.99) |
| Trouble seeing even with glass/contacts | 3 (0.05) |
| BMI | 112 (1.81) |
| TC | 284 (4.59) |
| Smoking status | 3 (0.05) |
| Drinking status | 320 (5.17) |

PIR: poverty-income ratio, BMI: body mass index, TC: total cholesterol
