# Supplementary material for: Association between dietary calcium, potassium, and magnesium consumption and glaucoma
Source: PLoS One. 2023 Oct 18;18(10):e0292883. doi: 10.1371/journal.pone.0292883 (PMC10584168; doi:10.1371/journal.pone.0292883)
Supplement: S2 Table — (DOCX) [file pone.0292883.s003.docx]

**Table S2. Sensitivity analysis of characteristics of participants before and after imputation of missing data**

| Variables | Before imputation (n=6189) | After imputation (n=6189) | Statistics | *P* |
| --- | --- | --- | --- | --- |
| PIR, ratio, M (Q_1_, Q_3_) | 2.42 (1.28, 4.50) | 2.39 (1.25, 4.48) | Z=0.224 | 0.823 |
| Education level, n (%) |  |  | χ^2^=0.001 | 1.000 |
| Less than 9th grade | 923 (14.92) | 923 (14.91) |  |  |
| 9-11th grade (Includes 12th grade with no diploma) | 952 (15.39) | 952 (15.38) |  |  |
| High school graduate/ GED or equivalent | 1524 (24.64) | 1526 (24.66) |  |  |
| Some college or AA degree | 1551 (25.08) | 1552 (25.08) |  |  |
| Less than 9th grade | 1235 (19.97) | 1236 (19.97) |  |  |
| Marital status, n (%) |  |  | χ^2^=0.001 | 1.000 |
| Married | 3632 (58.71) | 3634 (58.72) |  |  |
| Widowed | 819 (13.24) | 819 (13.23) |  |  |
| Divorced | 854 (13.81) | 854 (13.80) |  |  |
| Separated | 221 (3.57) | 221 (3.57) |  |  |
| Never married | 415 (6.71) | 416 (6.72) |  |  |
| Living with partner | 245 (3.96) | 245 (3.96) |  |  |
| Drinking status, n (%) |  |  | χ^2^=0.002 | 0.999 |
| Frequently | 1293 (22.03) | 1363 (22.02) |  |  |
| Occasionally | 2684 (45.73) | 2833 (45.77) |  |  |
| Never | 1892 (32.24) | 1993 (32.20) |  |  |
| Smoking status, n (%) |  |  | χ^2^=0.001 | 1.000 |
| Yes | 1194 (19.30) | 1194 (19.29) |  |  |
| No | 2999 (48.48) | 3002 (48.51) |  |  |
| Quitted | 1993 (32.22) | 1993 (32.20) |  |  |
| Eye surgery for nearsightedness, n (%) |  |  | χ^2^=0.020 | 0.886 |
| Yes | 131 (2.21) | 139 (2.25) |  |  |
| No | 5803 (97.79) | 6050 (97.75) |  |  |
| Eye surgery for cataracts, n (%) |  |  | χ^2^=0.015 | 0.904 |
| Yes | 802 (13.50) | 840 (13.57) |  |  |
| No | 5140 (86.50) | 5349 (86.43) |  |  |
| Trouble seeing even with glass/contacts, n (%) |  |  | χ^2^=0.001 | 0.979 |
| Yes | 1540 (24.89) | 1542 (24.92) |  |  |
| No | 4646 (75.11) | 4647 (75.08) |  |  |
| BMI, kg/m^2^, Mean±SD | 29.19 ± 6.47 | 29.19 ± 6.47 | t=-0.02 | 0.984 |
| TC, mg/dL, Mean±SD | 201.48 ± 42.46 | 201.53 ± 42.51 | t=-0.07 | 0.945 |

t: test, Z: rank sum test, χ^2^: chi-square test

M: median, Q1:1st quartile, Q3:3rd quartile, BMI: body mass index, SD: standard deviation, TC: total cholesterol
