# Supplementary material for: Association between dietary calcium, potassium, and magnesium consumption and glaucoma
Source: PLoS One. 2023 Oct 18;18(10):e0292883. doi: 10.1371/journal.pone.0292883 (PMC10584168; doi:10.1371/journal.pone.0292883)
Supplement: S3 Table — (DOCX) [file pone.0292883.s004.docx]

**Table S3. Covariates of glaucoma**

| Variables | OR (95% CI) | *P* |
| --- | --- | --- |
| Age | 1.06 (1.05-1.08) | <0.001 |
| Gender |  |  |
| Male | Ref |  |
| Female | 0.90 (0.68-1.20) | 0.480 |
| Race |  |  |
| Mexican American | Ref |  |
| Non-Hispanic White | 1.29 (0.83-2.00) | 0.243 |
| Non-Hispanic Black | 2.31 (1.45-3.66) | <0.001 |
| Other Hispanic | 1.08 (0.50-2.36) | 0.836 |
| Other Race - Including Multi-Racial | 1.46 (0.66-3.22) | 0.335 |
| PIR | 0.88 (0.81-0.95) | 0.003 |
| Education level |  |  |
| Less than 9th grade | Ref |  |
| 9-11th grade (Includes 12th grade with no diploma) | 0.58 (0.37-0.91) | 0.020 |
| High school graduate/ GED or Equivalent | 0.64 (0.40-1.03) | 0.065 |
| Some college or AA degree | 0.54 (0.34-0.84) | 0.009 |
| College graduate or above | 0.34 (0.22-0.53) | <0.001 |
| Marital status |  |  |
| Married | Ref |  |
| Widowed | 3.33 (2.48-4.48) | <0.001 |
| Divorced | 0.97 (0.68-1.37) | 0.839 |
| Separated | 1.50 (0.70-3.24) | 0.289 |
| Never married | 1.34 (0.63-2.85) | 0.432 |
| Living with partner | 0.75 (0.26-2.20) | 0.593 |
| Drinking status |  |  |
| Frequently | Ref |  |
| Occasionally | 0.99 (0.67-1.47) | 0.972 |
| Never | 1.15 (0.81-1.63) | 0.409 |
| Smoking status |  |  |
| Yes | Ref |  |
| No | 1.36 (0.82-2.26) | 0.230 |
| Quitted | 2.01 (1.21-3.33) | 0.008 |
| Physical activity |  |  |
| <450 | Ref |  |
| ≥450 | 0.64 (0.50-0.81) | <0.001 |
| Screen time |  |  |
| Not long | Ref |  |
| Long time | 1.64 (1.13-2.39) | 0.011 |
| Unknown | 1.17 (0.88-1.55) | 0.278 |
| Eye surgery for nearsightedness |  |  |
| Yes | Ref |  |
| No | 0.51 (0.21-1.20) | 0.117 |
| Eye surgery for cataracts |  |  |
| Yes | Ref |  |
| No | 0.29 (0.21-0.40) | <0.001 |
| Trouble seeing even with glass/contacts |  |  |
| Yes | Ref |  |
| No | 0.43 (0.31-0.60) | <0.001 |
| Diabetes |  |  |
| No | Ref |  |
| Yes | 2.34 (1.84-2.97) | <0.001 |
| BMI | 0.99 (0.97-1.01) | 0.162 |
| TC | 0.99 (0.99-0.99) | <0.001 |
| Usage of β-adrenergic blocking agents |  |  |
| No | Ref |  |
| Yes | 1.77 (1.35-2.33) | <0.001 |
| Energy intake | 0.99 (0.99-0.99) | <0.001 |

OR: odds ratio, CI: confidence interval, Ref: reference, PIR: poverty-income ratio, BMI: body mass index, TC: total cholesterol
