# Supplementary material for: Association between dietary calcium, potassium, and magnesium consumption and glaucoma
Source: PLoS One. 2023 Oct 18;18(10):e0292883. doi: 10.1371/journal.pone.0292883 (PMC10584168; doi:10.1371/journal.pone.0292883)
Supplement: S4 Table — (DOCX) [file pone.0292883.s005.docx]

**Table S4. Covariates of glaucoma in hypertension/non-hypertension persons**

| Variables | Non-hypertension  (n=2418) | | Hypertension  (n=3771) | |
| --- | --- | --- | --- | --- |
|  | OR (95% CI) | *P* | OR (95% CI) | *P* |
| Age | 1.06 (1.02-1.09) | <0.001 | 1.06 (1.04-1.08) | <0.001 |
| Gender |  |  |  |  |
| Male | Ref |  | Ref |  |
| Female | 0.86 (0.48-1.52) | 0.586 | 0.90 (0.64-1.27) | 0.549 |
| Race |  |  |  |  |
| Mexican American | Ref |  | Ref |  |
| Non-Hispanic White | 2.36 (1.13-4.91) | 0.023 | 0.85 (0.55-1.32) | 0.465 |
| Non-Hispanic Black | 4.83 (2.40-9.76) | <0.001 | 1.34 (0.82-2.21) | 0.235 |
| Other Hispanic | 1.79 (0.44-7.39) | 0.406 | 0.76 (0.33-1.80) | 0.526 |
| Other Race - Including Multi-Racial | 1.17 (0.29-4.70) | 0.825 | 1.23 (0.51-2.94) | 0.640 |
| PIR | 0.88 (0.75-1.03) | 0.115 | 0.92 (0.83-1.02) | 0.122 |
| Education level |  |  |  |  |
| Less than 9th grade | Ref |  | Ref |  |
| 9-11th grade (Includes 12th grade with no diploma) | 0.30 (0.12-0.73) | 0.010 | 0.70 (0.44-1.10) | 0.119 |
| High school graduate/ GED or Equivalent | 0.48 (0.17-1.32) | 0.147 | 0.75 (0.45-1.24) | 0.254 |
| Some college or AA degree | 0.49 (0.19-1.25) | 0.132 | 0.63 (0.43-0.93) | 0.022 |
| College graduate or above | 0.24 (0.09-0.64) | 0.006 | 0.46 (0.27-0.79) | 0.006 |
| Marital status |  |  |  |  |
| Married | Ref |  | Ref |  |
| Widowed | 3.39 (1.66-6.94) | 0.002 | 2.64 (1.75-4.00) | <0.001 |
| Divorced | 0.71 (0.32-1.60) | 0.400 | 1.03 (0.67-1.60) | 0.887 |
| Separated | 0.52 (0.07-4.19) | 0.530 | 1.70 (0.71-4.08) | 0.223 |
| Never married | 2.18 (0.52-9.09) | 0.274 | 1.04 (0.53-2.05) | 0.906 |
| Living with partner | 1.75 (0.46-6.61) | 0.395 | 0.29 (0.10-0.83) | 0.023 |
| Drinking status |  |  |  |  |
| Frequently | Ref |  | Ref |  |
| Occasionally | 1.30 (0.59-2.86) | 0.508 | 0.83 (0.53-1.30) | 0.402 |
| Never | 1.01 (0.45-2.29) | 0.979 | 1.01 (0.69-1.49) | 0.950 |
| Smoking status |  |  |  |  |
| Yes | Ref |  | Ref |  |
| No | 0.88 (0.34-2.33) | 0.796 | 1.54 (0.91-2.61) | 0.108 |
| Quitted | 1.69 (0.61-4.69) | 0.303 | 1.98 (1.22-3.22) | 0.007 |
| Physical activity |  |  |  |  |
| <450 | Ref |  | Ref |  |
| ≥450 | 0.65 (0.36-1.20) | 0.163 | 0.71 (0.52-0.95) | 0.024 |
| Screen time |  |  |  |  |
| Not long | Ref |  | Ref |  |
| Long time | 2.02 (0.79-5.15) | 0.136 | 1.24 (0.87-1.76) | 0.231 |
| Unknown | 1.15 (0.59-2.22) | 0.677 | 1.04 (0.71-1.52) | 0.843 |
| Eye surgery for nearsightedness |  |  |  |  |
| Yes | Ref |  | Ref |  |
| No | 0.18 (0.05-0.60) | 0.007 | 1.10 (0.39-3.12) | 0.857 |
| Eye surgery for cataracts |  |  |  |  |
| Yes | Ref |  | Ref |  |
| No | 0.28 (0.15-0.51) | <0.001 | 0.37 (0.26-0.52) | <0.001 |
| Trouble seeing even with glass/contacts |  |  |  |  |
| Yes | Ref |  | Ref |  |
| No | 0.39 (0.18-0.85) | 0.019 | 0.47 (0.32-0.67) | <0.001 |
| Diabetes |  |  |  |  |
| No | Ref |  | Ref |  |
| Yes | 2.15 (1.16-3.95) | 0.016 | 1.83 (1.46-2.30) | <0.001 |
| BMI | 0.96 (0.90-1.02) | 0.208 | 0.98 (0.96-0.99) | 0.012 |
| TC | 1.00 (0.99-1.01) | 0.934 | 0.99 (0.99-0.99) | <0.001 |
| Usage of β-adrenergic blocking agents |  |  |  |  |
| No |  |  | Ref |  |
| Yes |  |  | 1.21 (0.90-1.64) | 0.201 |
| Energy intake | 1.00 (1.00-0.99) | 0.004 | 1.00 (1.00-0.99) | <0.001 |

OR: odds ratio, CI: confidence interval, Ref: reference, PIR: poverty-income ratio, BMI: body mass index, TC: total cholesterol
