# Supplementary material for: Association between dietary calcium, potassium, and magnesium consumption and glaucoma
Source: PLoS One. 2023 Oct 18;18(10):e0292883. doi: 10.1371/journal.pone.0292883 (PMC10584168; doi:10.1371/journal.pone.0292883)
Supplement: Attachment — Submitted filename: Response to reviewers comments.docx [file pone.0292883.s006.docx]

**Responses to Reviewers**

Dear Editor and reviewers,

We highly appreciate and thank the Editors and the reviewers for your time and valuable comments on this manuscript. We have carefully studied the comments and suggestions and revised our paper accordingly. The following are our point-by-point responses to the comments. We hope that the revisions are acceptable and that our responses adequately address the comments. Thank you for your consideration.

**Responses to Reviewer #1’s comments**

Reviewer #1: Zhang et al. investigated the association between dietary Ca, K, and Mg consumption and glaucoma. I have only one minor question.

A Canon nonmydriatic camera was used to obtain the retinal image in the current study. Were all retinal images clear to assess vCDR? Older subjects often have lens opacity or miotic eye. These eye conditions often prevent to obtain clear retinal image.

Response: Thank you for your commont. The methods to assess vCDR were the standard methods provided in the NHANES database. The retinal image of eyes was evaluated by at least two of three professional eye doctors, and we belive that the eligible retinal images were all clear to assess vCDR.
